# Electronic transfusion consent and blood delivering pattern improve the management of blood bank in China

**DOI:** 10.1186/s12913-022-07825-6

**Published:** 2022-04-26

**Authors:** Luxi Jiang, Guobing Zhang, Ke Hao, Weiling Xiang, Qin Zhang, Yiwei Xie, Zhen Wang, Bingyu Chen, Yaoqiang Du

**Affiliations:** 1 Laboratory Medicine Center, Department of Transfusion Medicine, Zhejiang Provincial People’s Hospital (Affiliated People’s Hospital, Hangzhou Medical College), No.158 Shangtang Road, Hangzhou, Zhejiang China; 2 XianJu People’s Hospital, Zhejiang Southeast Campus of Zhejiang Provincial People’s Hospital, No.53 Chengbei East Road, Xianju, Zhejiang China; 3 Department of Quality Management, Zhejiang Provincial People’s Hospital (Affiliated People’s Hospital, Hangzhou Medical College), No.158 Shangtang Road, Hangzhou, Zhejiang China

**Keywords:** Electronic transfusion consent, Blood delivering, Transfusion management system, Labor cost

## Abstract

**Background:**

The aim of this study was to improve the blood transfusion treatment consent accuracy, simplify the verification process, prolong the temperature control time before the blood transfusion, and save the blood transportation labor cost.

**Methods:**

We designed the blood transfusion consent electronic signing process, which can generate personalized the text content and can automatically check the filling accuracy. The signal can be transmitted to the blood transfusion management system (TMS) to relieving the blood distribution. For blood delivering pattern, we established the blood transport center, recruited full-time nurses and used temperature-controlled blood transfer boxes to deliver blood in batches on a regular basis.

**Results:**

A quarterly data analysis of blood transfusion quality showed a 100% blood transfusion consent accuracy after an electronic signing process was implemented. The average confirmation time savings between the electronic content and paper content was 26 min for the Department of Emergency (estimated difference 95% CI = 26 (20 to 36), *p* < 0.05). The blood delivering pattern reduced the time for each unit by leaving the average temperature control by 7.24 min (estimated difference 95% CI = 7.24 (6.92 to 7.56), *p* < 0.05). Furthermore, $3.67 was saved for the blood transportation labor cost for each unit as well.

**Conclusion:**

Blood transfusion consent electronic signing process not only ensures the accuracy, but also saves the verification time. Moreover, the blood delivering pattern prolongs the blood temperature control time and saves blood transportation labor costs. Thus, these two improvements could enhance transfusion management.

**Supplementary Information:**

The online version contains supplementary material available at 10.1186/s12913-022-07825-6.

## Background

Blood transfusion is a major lifesaving frontline procedure in majority of the clinical wards [1]. According to the *Annual SHOT Report 2019*, the main cause of the majority of serious hazard incidents are blood transfusion errors, accounting for up to 84.1% (2857/3397) [2]. Quality management is the key to a safe blood transfusion [3]. In China, the blood transfusion process starts with a doctor’s assessment of the patient’s condition, the patient is informed and signs the blood transfusion consent, and then the doctor applies the blood transfusion. For nurses, they need to collect the specimens, provide a specimen distribution notice, receive the blood that has been confirmed by the crossmatching tests, then transfuse and monitor blood transfusion reactions [4]. After the blood transfusion, the doctors and nurses need to finish the posttransfusion evaluation and update the medical records. The blood transfusion process is complicated. It involves the interaction between doctors, nurses, blood bank staffs, and caregivers. They also need to comply with all the laws and regulations [5].

In 2020, the Advisory Committee on the Safety of Blood, Tissues and Organs (SaBTO) updated the recommendations on the patient’s blood transfusion consent, and supersede the previous SaBTO ‘Patient consent for blood transfusion guidelines’ (October 2011) [6, 7]. Patients should be fully informed of the blood transfusion reasons, benefits, and risks alternatives. Moreover, patients should also give their blood transfusion consent [7]. According to the *Technical Specifications for Clinical Blood Transfusion adopted by the Ministry of Health of the People's Republic of China*, the doctors should explain to the patient or his/her family members the allogeneic blood transfusion adverse reactions and the possibility of blood-transmitted diseases, obtain the patient’s or his/her family member’s blood transfusion consent, and sign on the blood transfusion treatment consent with patients when deciding the blood transfusion treatment [8]. Furthermore, the doctors should put the form in the patient’s medical record. This provision has become a monitoring indicator for evaluating the blood transfusion management and hospital management [9]. However, it is hard to reach a 100% blood transfusion management process.

After the blood unit left the blood bank, the transportation management and temperature control management are encountered problems in the blood transfusion process management [10]. According to the *Technical Specifications for Clinical Blood Transfusion*, the medical staff (doctors and nurses) should go to the blood bank to collect the units after the blood crossmatching test [8]. They need to check the blood information with the blood bank staff. Once the blood unit left the blood bank, the return of the blood unit is not allowed [8]. The doctors and nurses in China are often very busy. It takes time and effort to get the blood unit back and forth, especially when waiting for the elevators. Due to the lack of temperature monitoring, the blood unit that left the blood bank’s temperature control cannot be returned for reuse [11, 12]. This also makes the staff very cautious about taking the blood unit, often carrying only one unit for a patient at a time and requiring a longer round trip.

**Fig. 1:**
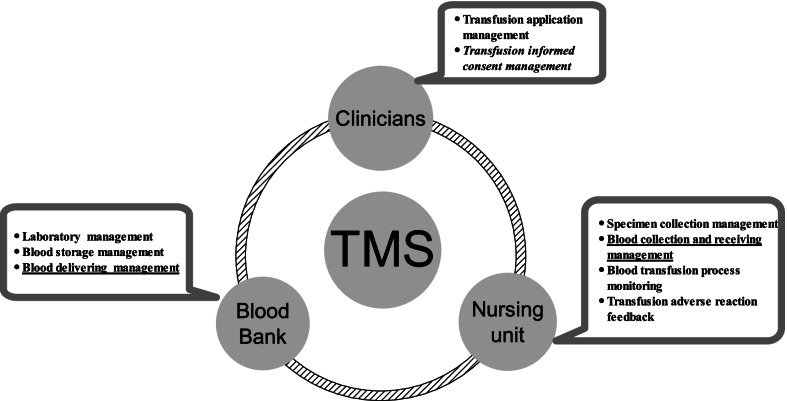
The TMS main frame in the hospital. The italic part was the quality project to be added. The underlined part was the quality project to be optimized

The hospital is a large, comprehensive, grade III class A hospital with 3,000-bed capacity in Zhejiang Province of China. The annual red blood cell dosage is about 16,000 U. On the other hand, the plasma dosage is about 2,300,000 ml. Since 2017, the blood transfusion management system (TMS) was introduced, and the whole blood transfusion electronic management basic flow was implemented. The information interconnection with the blood service institutions was also realized outside the hospital. The blood transfusion electronic management in the hospital was divided into three ports as follows: one each for blood bank, clinicians and nursing unit. They managed different blood transfusion procedures and exchanged the procedure data. The hospital’s TMS main frame is shown in Fig. 1. In order to improve the blood transfusion management and speed up the blood transfusion process efficiency, we tried to add the electronic management of informed consent for blood transfusion and optimize blood delivering process in the hospital.

## Methods

### Data collection

The study was conducted in June 2019 to December 2019. All the blood transfusion process management data of the patients were collected for 7 months from the hospital information system (HIS) and TMS, including the blood transfusion application time, the paper consent delivery time, the electronic consent signing time, the blood receipt printing time by the ward nurse, the blood distribution sending time, the blood leaving time, the blood checking time, the blood infusion time, etc. The data before and after the improvement were all involved. We also collected staff salary information. The ethical approval and participation consent were supported by the Ethics Committee for Zhejiang Provincial People’s Hospital in Hangzhou, Zhejiang, China (2022QT007).

### Optimize the consent verification process using electronic transfusion consent

Based on the certificate authority certification technology, the hospital achieved a paperless medical record management. We designed the blood transfusion treatment consent electronic signing process which covered the paper consent contents, including basic patient information, laboratory data, current transfusion evaluation, transfusion risk, and alternative treatments such as autotransfusion. We also added optional special circumstances, such as the emergency mismatched transfusion risks of a rare blood type, and carriers of drug antibodies, autoantibodies or isoantibodies (Additional file 1). When the doctors opened the patients’ blood transfusion treatment consent forms, the system automatically extracted the data and generated pre-printed form document. The doctors could choose the optional special circumstance, and the blood transfusion consent would increase the corresponding risk text. The doctors had to explain the content of the blood transfusion consent form to the patients or his/her family members, obtained their blood transfusion consent, and jointly signed the document with an electronic tablet with patients or his/her family members. Once the doctor saved the file, the electronic blood transfusion consent document was generated. It was automatically kept in the electronic medical record, and was transmitted to the TMS through the signals. This process saved the doctors a lot of work.

**Fig. 2:**
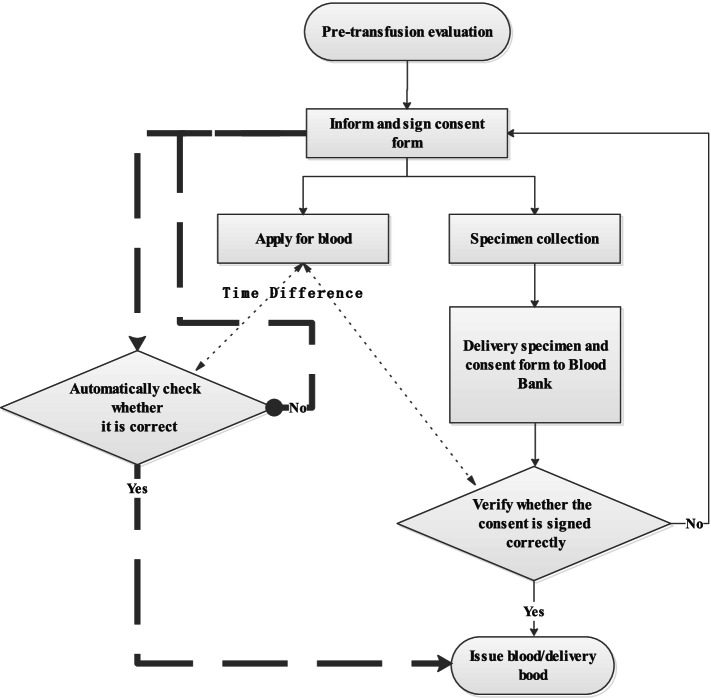
The comparison between the flow of electronic blood transfusion consent and paper blood transfusion consent process. The thickness of lines and arrows was representing an improved electronic blood transfusion consent process. The double arrow dotted line path represents the two ways of calculating the time spent for blood transfusion consent confirmation

Prior to the introduction of electronic blood transfusion consent forms, the patients signed the paper blood transfusion consent forms, and the caregivers need to deliver the forms to the blood bank. The staffs checked whether the blood transfusion consent forms were signed correctly, and then they released the blood after the confirmation. We completed the signing requirement at 100%, but it took a lot of time and manpower. The electronic blood transfusion consent flow was quite different from the previous paper forms, which did not need to be delivered to the blood bank after it was signed. It had an automatic verification function. If the electronic blood transfusion consent form was not filled with the standard format or it was incomplete, it would not be saved and thus required correction. If it was already corrected, the file would generate a confirmation message and would transmit it to the TMS. The blood distribution was no longer blocked when the TMS obtained the message. The comparison between the electronic blood transfusion consent and paper blood transfusion consent flow is illustrated in Fig. 2.

### Optimize the blood delivering pattern from blood bank to ward

#### Establish a blood transport center to provide full-time blood distribution services

The prior way of taking blood was very time-consuming. The blood bank staff reviewed the experimental results and uploaded the blood pre-outbound information to the TMS. After receiving the information from the TMS, the ward nurses selected the blood to be transfused according to the patient's condition and printed the blood receipt. They need to bring the receipts and boxes to the blood bank, checked the blood units with the staff, took the blood units back to the ward, examined again, and prepared for blood transfusion. Due to the existing blood transfusion regulations in China, and lack of the blood temperature monitoring, the medical staff usually took only one bag for patient’s blood unit at a time. Medical staff often complained that the prior way was a waste of time, especially in high-floor wards, where they had to wait extra time for lifts.

**Fig. 3:**
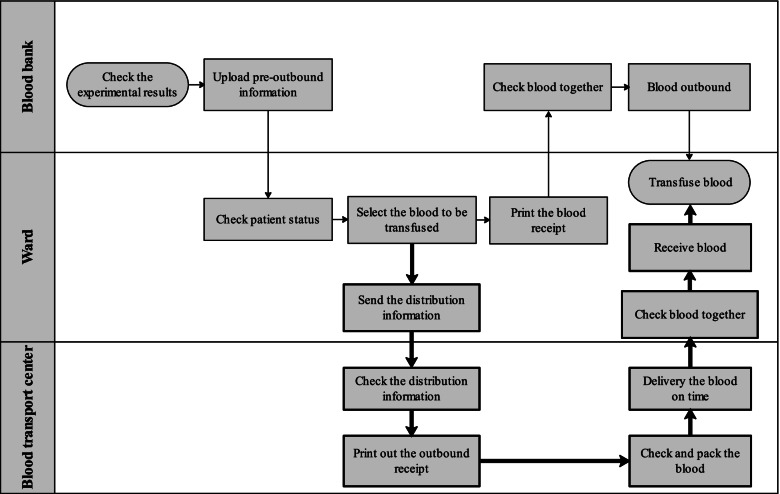
Flowcharts of the two ways of blood transport process. The thickness of lines and borders was representing the blood delivering pattern

Our hospital set up an in-hospital blood transport center in October 2019. The center was located in the blood bank and was equipped with five nurses who were responsible for blood distribution throughout the hospital. The blood distribution process followed this flow: The ward nurse selected the blood information to be transfused, and they sent the distribution information. The nurses in the blood transport center checked the blood information, printed out the outbound receipt, completed the blood unit outbound check, and packed and delivered the blood units to the ward on time with the specific blood transfer boxes. They checked and handed over blood units in the ward with the ward nurses. After which, they recorded it in the TMS (Partial screenshots of blood delivering process is illustrated in Additional file 2). When the patient needed a blood transfusion, the ward nurses took the blood unit from the transfer boxes, which had temperature monitoring, and rewarmed it for blood transfusion in a room temperature. There was always a nurse in charge of delivering emergency blood units for operating rooms and emergencies. The flowcharts of the two ways of blood unit transport are illustrated in Fig. 3. We still reserved the doctor’s and ward nurse’s paths going to the blood bank by themselves to take blood units for emergency rescue.

#### Replace the blood transfer box, realize the whole blood temperature control coverage

Before we implemented the improvement, once the blood unit left the blood bank, it went off the temperature monitor. The blood unit was not allowed to be returned for reuse [8]. In order to extend the blood temperature control to the bedsides of the patients, we replaced the blood transfer boxes and implemented the temperature control information system management. According to the current technical specifications, the patient’s blood infusion sequence was the following: platelet, cryoprecipitate, plasma, and finally red blood cells. The work mainly focused on prolonging the red blood cells and plasma temperature monitoring. The transfer boxes in the hospital were divided into two types as follows: one was to use cold storage agent to keep low temperature (suitable for the red blood cells and plasma transport) and the other was to use in room temperature (suitable for the platelet and cryoprecipitate transport). A visual temperature monitoring interface was set outside the box, which can read the temperature inside the box in real time and uploaded the temperature to the temperature control information system every 10 min. Temperature control information system was equipped with a warning device. Once the temperature exceeded, the transfer box and temperature control information system would immediately alarm. These types of transfer boxes were mainly used for red blood cells and plasma delivering in hospital or red blood cells and plasma storage in ward of less than 1 h. We also had blood storage tanks that can be connected to the power supply, just like a refrigerator with refrigeration device, which could store red blood cells and plasma that cannot be transfused temporarily in the area for a longer time. In order to ensure the blood use permission, the transfer boxes were equipped with a two-dimensional combination lock. Therefore, authorized mobile phones can scan the code to open it.

### Quality data collection

#### Time difference between blood application and consent verification

The monthly transfusion quality data were collected, including the blood transfusion consent form accuracy rate. To evaluate the process improvement effect, we also calculated the time difference between the blood transfusion application and the blood distribution consent confirmation in the emergency department from June 2019 to December 2019. The calculation formulas were as follows:

Time difference of paper blood transfusion consent = time when the blood transfusion consent form was verified by the blood bank staff—blood application time.

Time difference of electronic blood transfusion consent = successful submission of electronic blood transfusion consent time—blood application time.

#### Time of blood leaving temperature control

The time of the blood unit leaving the temperature control before the blood transfusion is an important quality parameter. In general, the blood unit needed to be rewarmed at a room temperature before the blood transfusion. The blood private storage in the ward is not allowed. We calculated the time of the blood unit leaving the temperature control as follows:

For issuing the blood unit,

Time of blood unit leaving temperature control = start time of blood transfusion—time of blood unit outbound by blood bank staff.

For delivering the blood unit,

Time of blood unit leaving temperature control = start time of blood transfusion—time of blood unit received by ward nurses before blood transfusion.

(Note: blood receiving means removal of blood from the temperature-controlled transfer boxes by ward nurses).

#### The labor cost between issuing and delivering the blood units

We recorded the staff time for issuing and delivering the blood units. For issuing the blood units, data were collected on the time taken for the ward nurses to perform a return journey to the blood bank. The time record was from the ward nurse to print the blood receipt to the ward nurse to return to the ward to check the blood information. For delivering the blood units, the nurses from the blood delivery center recorded the time from printing the blood receipt of a delivering request to the time the blood unit was checked by the ward nurses at the ward. The labor cost for the blood transportation was calculated using the average time taken to perform multiplied by the hourly cost for the staff grade.

### Statistical analysis

A statistical analysis was performed by SPSS 22.0 software package. Because the data were skewed, the measurement data were expressed as Median (P25, P75). The two groups of data were compared based on the Mann–Whitney U test. Multiple comparisons were based on the Kruska-Wallis method. The *p*-value of < 0.05 was considered to be statistically significant.

## Results

### Electronic blood transfusion consent process ensured the accuracy and saved confirmation time

**Fig. 4:**
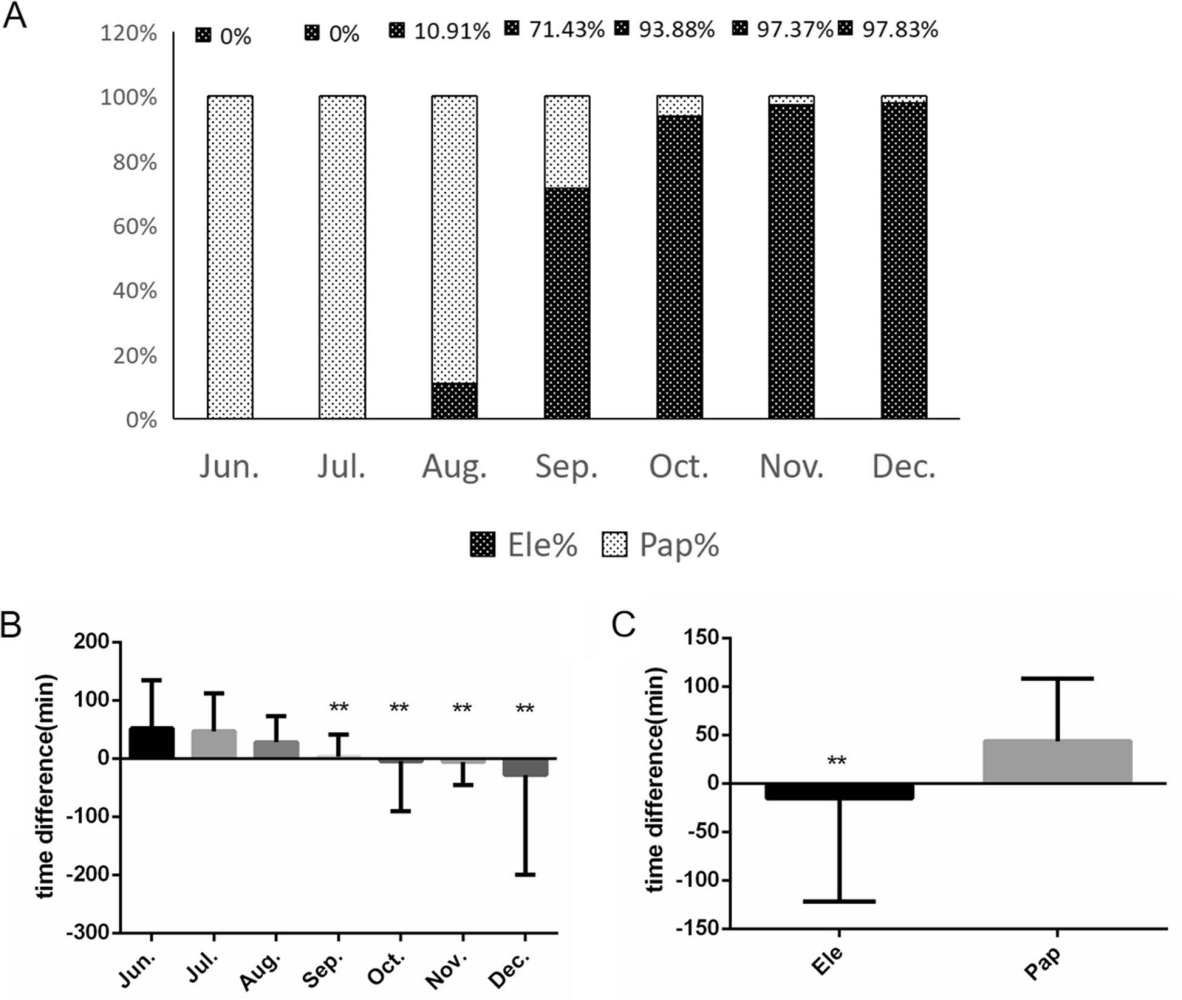
The changes in blood transfusion informed consent form and blood transfusion consent confirmation time. **A** Proportion of electronic and paper blood transfusion consent forms from June 2019 to December 2019. Ele: electronic, Pap: paper. **B** The time difference between blood transfusion consent confirmation and blood transfusion application in the emergency department from June 2019 to December 2019. Compared with June 2019, July 2019 and August 2019, the difference was significant (**: *p* < 0.001). **C** The time difference of electronic blood transfusion and paper blood transfusion consents. The difference was significant (**: *p* < 0.001)

We gradually introduced electronic blood transfusion consents from August 2019. It accounted for more than 97% from November 2019 to December 2019 (Fig. 4A). A valid blood transfusion consent was obtained and documented for each blood transfusion during electronic blood transfusion consent implementation because of the system’s interception. A data quarterly analysis of blood transfusion quality showed that the blood transfusion consent accuracy was 100%.

We also analyzed the time difference between blood transfusion consent form confirmation and blood transfusion application in the emergency department every month. The time difference was significantly reduced since September 2019 when the ratio of electronic blood transfusion consent forms was 71.43% (Fig. 4B). The average electronic time difference was negative, because blood transfusion consent forms were always signed before the application for blood transfusion. Furthermore, the electronic blood transfusion consent forms were confirmed automatically by the system. There was no delay in the emergency blood distribution. The time differences of all electronic blood transfusion consent forms and paper blood transfusion consent forms were compared (Table 1, Additional file 3). The results showed that electronic blood transfusion consent forms save the confirmation time significantly compared to paper blood transfusion consent forms. The average time savings was 26 min (Fig. 4C).

**Table 1:** The time difference of paper blood transfusion and electronic blood transfusion consent process

Item	M (P25, P75)	Estimated Difference 95% (CI)	Mann–Whitney U test	
			Z value	*p* value
Paper	21 (12, 53)	26 (20 to 36)	-11.398	< 0.001
Electronic	2 (-4.75, 8.80)			

### Delivering blood pattern increased blood temperature control time

**Fig. 5:**
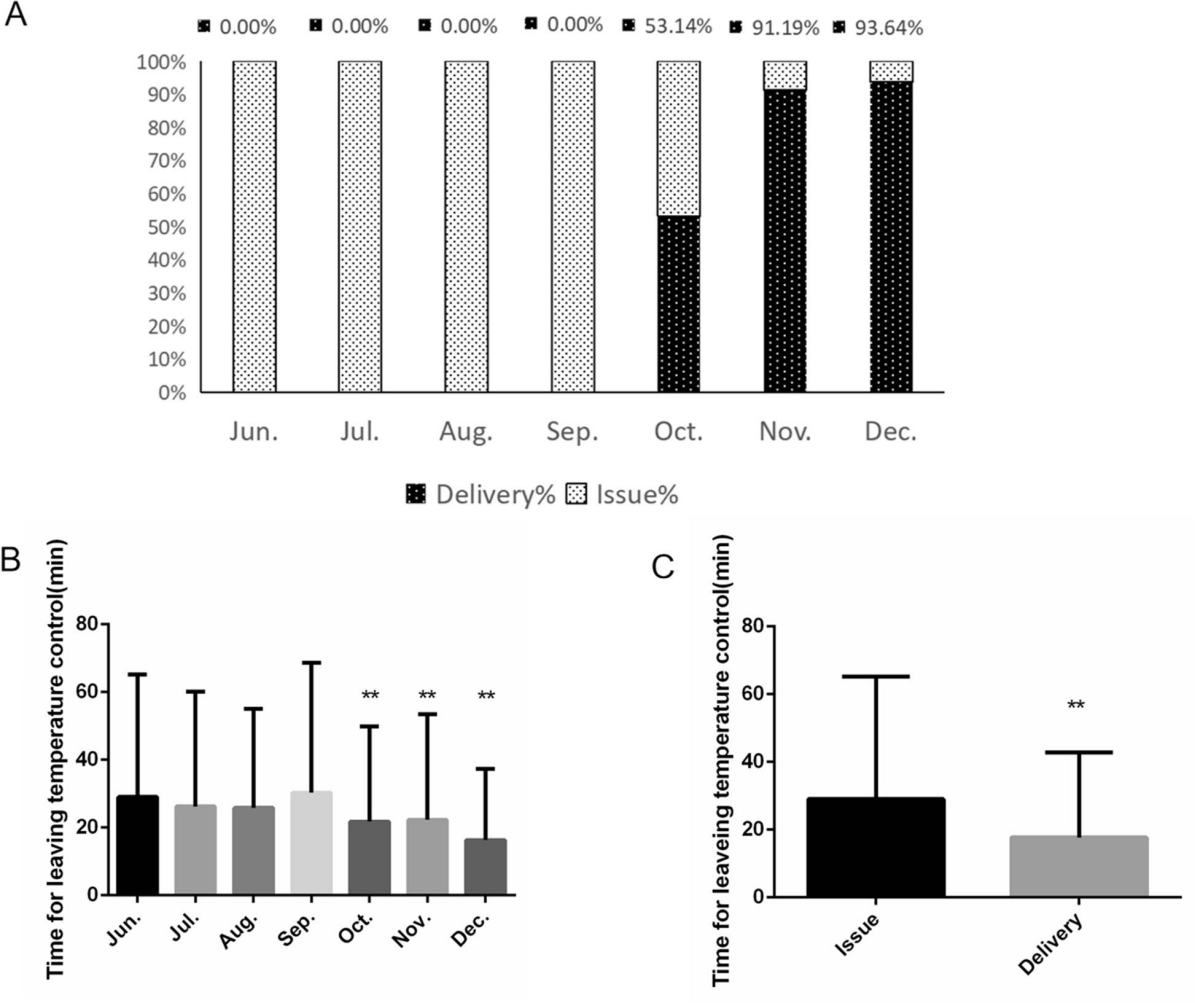
The changes of blood transport pattern and temperature control time. **A** Proportion of different blood transport modes from June 2019 to December 2019. **B** The time for blood unit leaving temperature control from June 2019 to December 2019. Compared with June 2019, July 2019, August 2019 and September 2019, the difference was significant (**: *p* < 0.001). **C** The leaving temperature control time between issuing and delivering blood. The difference was significant (**: *p* < 0.001)

We established the blood transport center in October 2019.The blood transport mode from the blood bank to the ward was changed, while the blood proportion delivered was 53.14% in October 2019 and 93.64% in December 2019 (Fig. 5A). We found that the average time for blood leaving temperature control was significantly reduced (Fig. 5B). We analyzed the blood temperature control time difference between the two modes of blood transportation (Table 2, Additional file 4). Delivering the blood units increased the temperature control time significantly (*p* < 0.05) (Fig. 5C).

**Table 2:** Leaving temperature control time of issuing and delivering the blood units

Item	M (P25, P75)	Estimated Difference 95% (CI)	Mann–Whitney U test	
			Z value	*p* value
Issuing	18.48 (12.12, 27.43)	7.24 (6.92 to 7.56)	-41.98	< 0.001
Delivering	10.53 (5.68, 18.91)			

### Delivering blood reduced the labor cost

We calculated the staff time of delivering and issuing for each blood unit. For delivering, the average time was 0.26 h; moreover, the issuing time was 0.62 h. The ward nurse time cost was $7.48/hour, while the blood delivery nurse time cost of who was delivering blood only was $3.74/hour. The staff time cost was saved by $3.67 for each blood unit. According to the hospital blood consumption in 2018 and 2019, the savings were estimated at $99,000 per year (Table 3).

**Table 3:** Cost comparison of the different modes of transporting blood units

Items	Issuing	Delivering
Average time for each unit (h)	0.62	0.26
Ward nurses time cost ($/h)	7.48	-
Transport center nurses time cost ($/h)	-	3.74
Total cost of staff time for each unit ($)	4.64	0.97
Annual saving in staff time costs that delivering blood contributed ($)	-	99,000^a^

*Note*: The exchange rate of $ (USD) to ¥ (CNY) here was 6.366 in December 2021; ^a^ Estimated Value

## Discussion

The blood transfusion safety concerns the life safety. Improving the blood transfusion management can improve the blood transfusion safety and effectiveness [13]. A number of clinical applications have benefited from the blood transfusion information management implementation or the blood transfusion information process improvement [14–16]. The hospital had implemented the blood transfusion electronic information management through the whole process since 2017. Since 2019, the Zhejiang Provincial Health Commission released “Without the Need for A Second Visit” reform for improving healthcare services [17], including the item of “No need to run for blood service.” The main purposes of the reform are making a full use of the Internet and big data to comprehensively promote good medical services. At the second half of 2019, the electronic blood transfusion consent process and the blood delivery process to complement the blood transfusion management were improved. Electronic blood transfusion consent not only guaranteed the signing accuracy, but also saved the confirmation turnaround time. Each blood transfusion consent form saved an average of 26 min. The blood delivering pattern reduced the time for each blood unit leaving temperature control by 7.24 min on average. Furthermore, the labor cost saved $3.67.

The patients should be fully informed before the blood transfusion [18]. Different countries have different policies and realities [18–22]. The lack of blood transfusion consent forms or lack of documentation which only had verbal blood transfusion consent forms remained to be a problem in some areas [19, 20]. This problem could be solved by using a pre-prepared paper blood transfusion consent form in a standard format and checking that the blood transfusion consent was completed before the blood transfusion [21, 22]. However, it also brought other problems. These problems could be classified into three categories as follows: 1. the paper blood transfusion consent form was incomplete [21, 23, 24]; 2. the content was outdated, the risks involved were not detailed enough, and the content of patient blood management was not reflected [22]; 3. low efficiency and manpower consumption [25].

The pre-printed blood transfusion consent forms were implemented before, but were plagued by these three problems. Therefore, the electronic blood transfusion treatment consent process was designed and operated, which can automatically extract patient laboratory test data, set standard format, automatically complete the format review, and refuse to save and submit the incomplete consents. According to the patient’s different conditions, the doctors can choose different risk content texts. The content of autologous transfusion is highlighted in bold and underlined font. Since the implementation of the electronic blood transfusion consent, blood transfusion consent filling accuracy rate was always 100% in quarterly blood transfusion quality sampling. This greatly saved the blood transfusion consent verification time and improved the efficiency as well. At present, the problem of “yes” was solved, how many patients had been effectively informed, and how many patients had truly understood the blood transfusion consent content. Paying attention to the participation in the decision is needed in the follow-up investigation [25]. A video animation and other forms will be more helpful for patients to understand the process [18].

Blood unit improper storage and transportation can result in blood wastage [26, 27]. Delayed infusion and improper storage after an issued blood unit also lead to higher blood transfusion adverse reactions [1]. Blood products temperature monitoring in hospitals is usually performed only in the blood bank for blood storage [11, 12, 27]. The blood units were in a state of unmonitored condition in clinical wards and theaters after the blood issued, especially red blood cells and plasma [1]. Therefore, extending the blood products temperature control to wards and theaters can improve blood transfusion safety. Electronic remote blood issue (ERBI) technology is thought to rely on TMS, automated refrigerators, and electronic crossmatching tests to enable remote self-service blood collection [28, 29]. The refrigerator is usually placed in the theaters or wards far away from the transfusion department to ensure the blood timely supply for emergency use [29]. This is also a way of extending temperature control to the ward. But it costs a fortune to equip each ward. Moreover, ERBI is not suitable for areas where blood resource is relatively scarce or electronic crossmatching tests have not been carried out. The blood transfer boxes were given more focus. For red blood cells and plasma, the blood transfer boxes can be refrigerated and can realize temperature monitoring and data transmission. According to the storage time, nurses could flexibly allocate the blood transfer boxes (cold storage agent or power supply) and realized the management of blood unit left from the blood bank. For platelet or cryoprecipitate, due to the shortage of these components and the blood transfusion sequence, it was not found that improper storage led to discarded blood in our previous blood transfusion quality inspection in ward, although the platelet and cryoprecipitate temperature control management through room temperature blood transfer boxes was achieved. However, considering the platelet storage condition complexity (appropriate amplitude) and the lack of precise platelet or cryoprecipitate temperature control, the platelet and cryoprecipitate ward storage was not achieved yet. For safety reasons, blood transfer boxes can be opened by scanning code. The nurses in the blood transport center delivered blood units with different types of blood transfer boxes according to the different needs of the ward. The blood unit temperature control time was significantly increased.

Another problem with blood transporting is the manpower cost [30]. In a study quantifying the cost of patient care with transfusion-dependent thalassemia, the procedure costs (55%) were higher than the blood costs (40%) [31]. In the United Kingdom, the nurses needed to collect samples, place requests for blood transfusion, and administer blood transfusion [32]. Specimen delivery and blood delivery were done by workers and cost only $9, but nursing inputs was still the most important part of the whole blood transfusion management chain [33]. In China, regulations clearly state that trained medical personnel (usually nurses) should be responsible for blood delivery [8]. This undoubtedly increases the nursing inputs. We changed the blood transport mode and replaced ward nurses with full-time blood delivering nurses. We delivered blood in batches on a regular basis and saved $3.67 per unit on average. The annual savings is also substantial, comparable to ERBI brings ($5,000 — $10,000) [33].

The benefits of electronic blood transfusion consent process and blood delivery model were reported in the hospital. Each hospital has different status and regulations, and it is unknown whether they apply to other hospitals. As previously described, the current electronic blood transfusion informed consent signing process was convenient, but it was uncertain if how many patients truly understood and participated. It is also unknown whether there was oversigning, which caused fatigue in patients or his/her family members [25]. The current blood delivery model was suitable for the anemia correction in clinical general conditions, but not for emergency treatment. There are accurate temperature control facilities for red blood cells and plasma at present, but there is none for platelet and cryoprecipitate. This is the direction of the future efforts. Due to the original regulations, blood unit which left the blood bank cannot be returned for reuse [8, 9]; therefore, the clinical blood withdrawal and scrapping situation was very rare, and it was impossible to compare the data at the present stage whether the blood temperature control coverage increase brings about the blood bag scrapping decrease.

## Conclusion

Blood transfusion is an important means of clinical treatment. The blood transfusion process information management can help standardize the blood transfusion process implementation and improve blood transfusion safety. We used information management technology to improve two blood transfusion processes as follow: electronic blood transfusion consent verification and blood delivery pattern. The automatic system check function not only ensured the blood transfusion consent filling accuracy, but also saved the manual check steps and speed up the blood transfusion process. A blood transport center was established and with the combination with the use of temperature-controlled blood transfer boxes, it solved the problem of temperature control after blood unit leaving the blood bank and also saved a considerable labor cost amount. It is recommended that there should be more transfusion management improvements to improve the transfusion safety in China.

## Supplementary Information

**Additional file 1.** Hospital Transfusion Treatment Consent.

**Additional file 2.** Partial screenshots of blood delivering process. **A** Interface of ward nurses to send blood delivering information. **B** Interface of full-time nurses to receive blood delivering information. **C** Interface of blood transfer between full-time nurses and ward nurses.

**Additional file 3.** Raw datasets of the consents. **A** Dataset of paper consents. **B** Dataset of electronic consents. **C** Total statistically proportion of paper and electronic consents per month (Excel file: sheets A, B and C).

**Additional file 4.** Raw data of blood transport pattern (Excel file).

## Data Availability

The datasets used and/or analyzed during the current study are available in the supplementary files and on reasonable request by corresponding or first authors.

## Abbreviations

SHOT: Serious hazards of transfusion
SaBTO: The Advisory Committee on the Safety of Blood, Tissues and Organs
Ele: Electronic content forms
Pap: Paper content forms
ERBI: Electronic remote blood issue
