# Supplementary material for: Electronic transfusion consent and blood delivering pattern improve the management of blood bank in China
Source: BMC Health Serv Res. 2022 Apr 26;22:561. doi: 10.1186/s12913-022-07825-6 (PMC9044836; doi:10.1186/s12913-022-07825-6)
Supplement: Supplementary file 1 — Additional file 1. Hospital Transfusion Treatment Consent. [file 12913_2022_7825_MOESM1_ESM.docx]

Hospital Transfusion Treatment Consent

Blood Group: ABO: RH:

Name: Gender: Age: Medical record No: Ward: Bed No:

Clinical diagnosis:

ALT U/L HBsAg HBeAg Anti-HBs

Anti-HBe Anti-HBc HCV

HIV-1/2 antibody+p24 antigen RPR TPHA

Blood transfusion purpose: Blood component:

Way of blood transfusion: Number of blood transfusion:

Blood transfusion therapy includes transfusion of whole blood and component blood, which is one of the important measures in clinical treatment and an effective means to rescue urgent and critically ill patients. Blood sources for blood transfusion therapy can be divided into autologous blood and allogeneic blood, but allogeneic blood transfusion has certain risks, which may lead to blood transfusion reaction and infection with blood-borne diseases.

Although the allogeneic blood used in our hospital has been tested in accordance with the relevant regulations of the Ministry of Health, due to the current limited level of science and technology, there are still some blood transfusion reactions and transfusion infectious diseases that cannot be predicted or prevented. The main situation that may happen when transfusion is as follows: **1. Allergic reaction, 2. Febrile reaction, 3. Infection with hepatitis B、C, etc, 4. Infection with AIDS, syphilis, 5. Infection with malaria, 6. Infection with cytomegalovirus or EB virus, 7. Other diseases caused by blood transfusion.**

**Autogenous blood infusion can avoid the risk of allogeneic blood, and patients with autogenous indications are encouraged to receive autogenous blood collection first.**

Remarks:

Here text selection can be made for a patient’s specific situation.

- Immediate transfusion is needed to save life, and serious adverse reactions may occur due to the presence of autoantibodies in the patient's serum.
- Immediate transfusion is needed to save life, and because the patient's Rh(D) blood type is negative, transfusion may produce unexpected antibodies.
- It is difficult to identify the patient's blood type, and immediate blood transfusion is needed to save life. Compatible infusion can be performed, and adverse blood transfusion reactions may occur.
- The patient's direct Coombs'test was positive, resulting in hemagglutination on the cross matching test. Immediate blood transfusion was required to save life, and adverse blood transfusion reactions may occur.
- Due to the presence of alloantibodies or the use of special drugs, blood type identification or cross matching test is incompatible, and immediate transfusion is needed to save life, and adverse blood transfusion reactions may occur.
- Risk of transfusion of apheresis platelets of different blood groups: antibodies in donor plasma may cause acute hemolysis or ineffective platelet transfusion.
- After hematopoietic stem cell transplantation, transfusion of type O washed red blood cells, type AB plasma and type AB platelets was required due to incompatibility of donor and recipient blood groups. However, due to the need of saving life, emergency transfusion of heterogeneous blood should be started immediately, which may cause adverse blood transfusion reactions.

Hemolytic transfusion reaction, irregular antibody generation and invalid infusion may occur after compatible transfusion; re-transfusion problems may occur after homogenous immune reaction in RhD negative patients; and the risk of neonatal hemolytic disease may occur after heterogeneous transfusion in female patients of childbearing age.

If you (your family/guardian) agree to receive blood transfusion during your stay in our hospital, please sign in the presence of the underwriter after you understand the above possible situations.

Patient’s opinion: (Please write agree or disagree) blood transfusion

Patient(family/guardian) signature: Time:

Clinician signature: Time:
