# Supplementary material for: Electronic transfusion consent and blood delivering pattern improve the management of blood bank in China
Source: BMC Health Serv Res. 2022 Apr 26;22:561. doi: 10.1186/s12913-022-07825-6 (PMC9044836; doi:10.1186/s12913-022-07825-6)
Supplement: Supplementary file 2 — Additional file 2. Partial screenshots of blood delivering process. A Interface of ward nurses to send blood delivering information. B Interface of full-time nurses to receive blood delivering information. C Interface of blood transfer between full-time nurses and ward nurses. [file 12913_2022_7825_MOESM2_ESM.pdf]

A

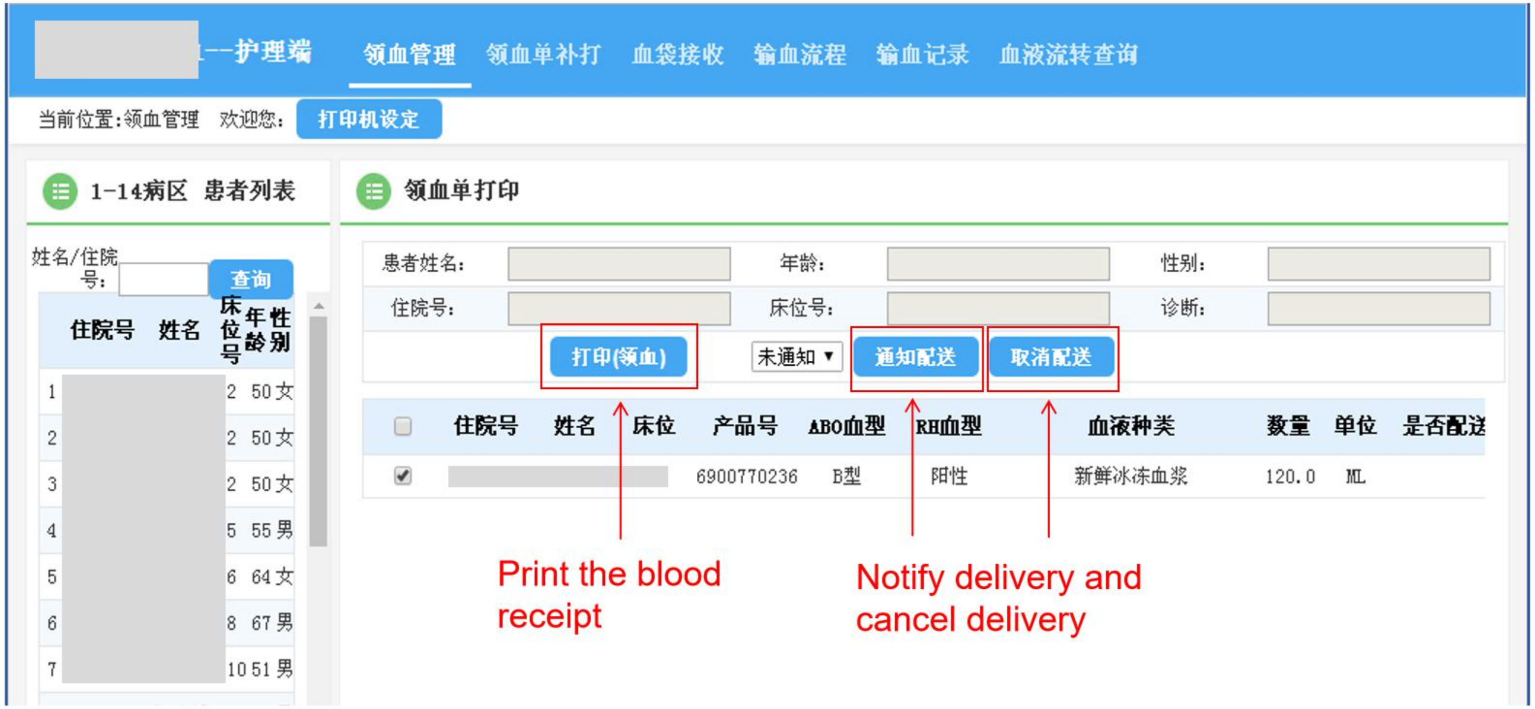
--护理端
领血管理
领血单补打
血袋接收
输血流程
输血记录
血液流转查询
当前位置:领血管理 欢迎您:
打印机设定
1-14病区 患者列表
姓名/住院号:
查询
住院号
姓名
床位号
年龄
性别
1 2 50 女
2 2 50 女
3 2 50 女
4 5 55 男
5 6 64 女
6 8 67 男
7 10 51 男
领血单打印
患者姓名:
年龄:
性别:
住院号:
床位号:
诊断:
打印(领血)
未通知
通知配送
取消配送
住院号
姓名
床位
产品号
ABO血型
RH血型
血液种类
数量
单位
是否配送
6900770236
B型
阳性
新鲜冰冻血浆
120.0
ML
Print the blood receipt
Notify delivery and cancel delivery

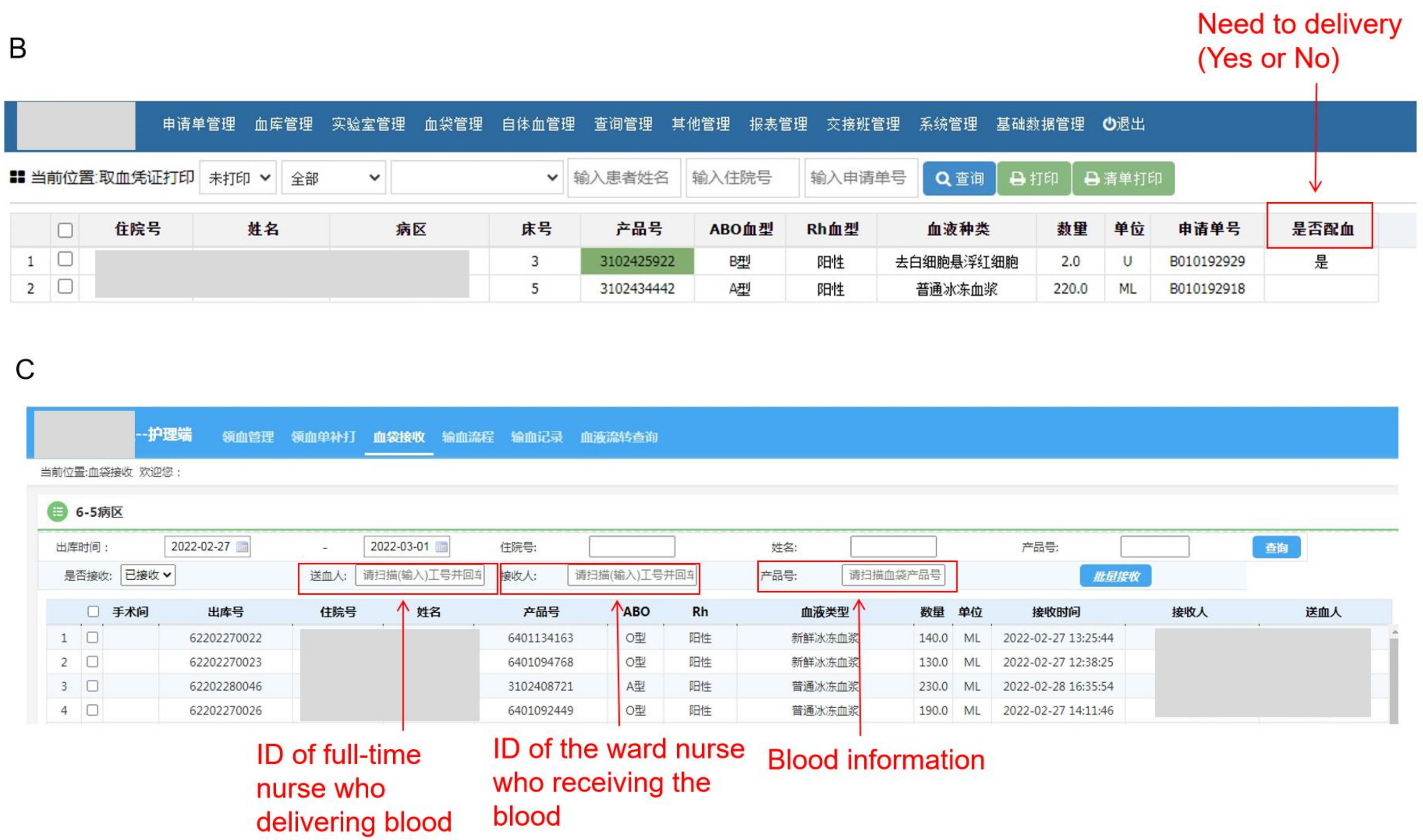
B
Need to delivery
(Yes or No)
申请单管理 血库管理 实验室管理 血袋管理 自体血管理 查询管理 其他管理 报表管理 交接班管理 系统管理 基础数据管理 退出
当前位置:取血凭证打印 未打印 全部 输入患者姓名 输入住院号 输入申请单号 查询 打印 清单打印
住院号 姓名 病区 床号 产品号 ABO血型 Rh血型 血液种类 数量 单位 申请单号 是否配血
1 3 3102425922 B型 阳性 去白细胞悬浮红细胞 2.0 U B010192929 是
2 5 3102434442 A型 阳性 普通冰冻血浆 220.0 ML B010192918
C
--护理端 领血管理 领血单补打 血袋接收 输血流程 输血记录 血液流转查询
当前位置:血袋接收 欢迎您：
6-5病区
出库时间： 2022-02-27 - 2022-03-01 住院号: 姓名: 产品号: 查询
是否接收: 已接收 送血人: 请扫描(输入)工号并回车 接收人: 请扫描(输入)工号并回车 产品号: 请扫描血袋产品号 批量接收
手术间 出库号 住院号 姓名 产品号 ABO Rh 血液类型 数量 单位 接收时间 接收人 送血人
1 62202270022 6401134163 O型 阳性 新鲜冰冻血浆 140.0 ML 2022-02-27 13:25:44
2 62202270023 6401094768 O型 阳性 新鲜冰冻血浆 130.0 ML 2022-02-27 12:38:25
3 62202280046 3102408721 A型 阳性 普通冰冻血浆 230.0 ML 2022-02-28 16:35:54
4 62202270026 6401092449 O型 阳性 普通冰冻血浆 190.0 ML 2022-02-27 14:11:46
ID of full-time nurse who delivering blood
ID of the ward nurse who receiving the blood
Blood information
